# County-level jail incarceration, community economic distress, rurality, and preterm birth among women in the US South

**DOI:** 10.1017/cts.2022.468

**Published:** 2022-10-28

**Authors:** Brooke E. E. Montgomery, George C. Pro, Don E. Willis, Nick D. Zaller

**Affiliations:** 1 Department of Health Behavior & Health Education, University of Arkansas for Medical Sciences, Fay W. Boozman College of Public Health, Little Rock, AR, USA; 2 University of Arkansas for Medical Sciences, Southern Public Health and Criminal Justice Research Center (S-PAC), Little Rock, AR, USA; 3 University of Arkansas for Medical Sciences Northwest, College of Medicine, Office of Community Health and Research, Little Rock, AR, USA

**Keywords:** Incarceration, distressed communities index, preterm birth, women’s health, rural health, reproductive health

## Abstract

**Introduction:**

The USA has higher rates of preterm birth and incarceration than any other developed nation, with rates of both being highest in Southern states and among Black Americans, potentially due to rurality and socioeconomic factors. To test our hypothesis that prior-year county-level rates of jail admission, economic distress, and rurality were positively associated with premature birth rates in the county of delivery in 2019 and that the strength of these associations is greater for Black women than for White or Hispanic women, we merged five datasets to perform multivariable analysis of data from 766 counties across 12 Southern/rural states.

**Methods:**

We used multivariable linear regression to model the percentage of babies born premature, stratified by Black (Model 1), Hispanic (Model 2), and White (Model 3) mothers. Each model included all three independent variables of interest measured using data from the Vera Institute, Distressed Communities Index, and Index of Relative Rurality.

**Results:**

In fully fitted stratified models, economic distress was positively associated with premature births among Black (*F* = 33.81, *p* < 0.0001) and White (*F* = 26.50, *p* < 0.0001) mothers. Rurality was associated with premature births among White mothers (*F* = 20.02, *p* < 0.0001). Jail admission rate was not associated with premature births among any racial group, and none of the study variables were associated with premature births among Hispanic mothers.

**Conclusions:**

Understanding the connections between preterm birth and enduring structural inequities is a necessary scientific endeavor to advance to later translational stages in health-disparities research

## Introduction

The USA continues to have the highest preterm birth rate (i.e., live birth before the completion of 37 weeks of gestation) of any high-income country [1]. In 2021, March of Dimes estimated that the US preterm birth rate was 10.1% [2], compared to an estimated global preterm birth rate of 10.6% in 2014 (most recent available) [3]. Although the US preterm birth rate is decreasing, Black women in the USA are still nearly 60% more likely to have a preterm birth than White women (approximately 14% compared to 9%) [2]. Preterm birth is the leading cause of mortality among Black infants in the USA and the second leading cause of infant mortality in the USA [4]. Preterm birth rate is used frequently to assess racial disparities in birth outcomes among US women because of its frequency in the population, its convincing linkage to stress-induced biophysiological pathways, and its strong association with other birth outcomes and the development of chronic conditions later in life [5,6].

Due to the severity of this public health crisis, the March of Dimes convened a multi-disciplinary group to develop a consensus statement on the causes of the Black–White disparity in preterm birth in the USA. The group identified 33 hypothesized causes and concluded that structural racism was the only upstream factor that fully explained unique epidemiologic findings in the Black–White disparity in preterm birth, including the lack of disparity between African-born Black women and US-born White women and the lack of protection against preterm birth afforded by educational and economic gains made by US-born Black women [7]. Fortunately, major public health institutions are increasingly promoting racial health equity. Campaigns such as the National Institutes of Health “Ending Structural Racism” initiative and the Centers for Disease Control and Prevention (CDC) declaration that “racism is a system” and a “serious threat to the public’s health” represent a much-needed shift in the national discourse around structural determinants of health.

Data from Southern and rural US states provide a unique shared context by which to study preterm birth and factors related to structural racism. Southern and rural states have some of the highest rates of preterm birth in the country [2] and have a shared agricultural and cultural history that supported the US slave economy and subsequent Jim Crow laws and mass incarceration policies [8]. Moreover, an unjust and racially biased criminal justice system is a key manifestation of structural racism that continues, especially in Southern states, to disproportionately burden Black populations and compromise their health [9,10]. Similar to preterm birth, the USA has the highest incarceration rate in the world (including state prisons, local jails, and other systems of confinement), and Southern states have the highest incarceration rate in the USA [1,2]. The number of incarcerated women in the USA increased by 475% between 1980 and 2020, and Southern states continue to have the highest numbers of incarcerated women [11]. In most states, the number of individuals with direct involvement in the criminal justice system (ICJS) (i.e., arrest, incarceration, probation, parole) has decreased steadily since peaking at nearly 7.4 million in 2007 [12], but ICJS rates continue to increase in many Southern states [13].

As for preterm birth outcomes for the USA and South, Black people in the nation and region are up to 5 times more likely than White people to be incarcerated [14,15]. Incarceration has significant consequences for generational health [16–18], highlighting the public health impact of indirect ICJS (i.e., a family member or spouse is incarcerated) – most incarcerated women have a child and are the primary caregiver [19–21]. Between 1991 and 2016 in the USA, the number of incarcerated women with children increased by 96%, and the number of incarcerated men with children increased nearly by 50% [20]. Even for non-incarcerated women, indirect ICJS may contribute to preterm birth through poverty, lack of support, and chronic stress [7,19,22].

Consequently, both direct and indirect ICJS can have health and socioeconomic consequences that increase the risk of preterm births and magnify existing racial disparities [20,23–25]. The hypothesized pathways are complex and multifactorial, spanning every socioecological level [26]. However, much of the existing research was conducted on the influence of individual- and interpersonal-level factors on preterm birth and related racial disparities [7]. Factors like neighborhood characteristics, environmental factors, and rurality are also hypothesized to increase risk of preterm births [7,27–29]. Rural residence is hypothesized to increase risk of preterm birth through contextual socioeconomic disadvantage, limited healthcare supply, and geographic isolation, but when controlled for these factors tend to attenuate, but not necessarily eliminate, the health disadvantage experienced by rural residents compared to their urban counterparts [30,31]. Similarly, the immigrant birthweight paradox, which is the epidemiologic finding that foreign-born women have lower rates of preterm birth compared to their US-born counterparts, is partially explained by residence in neighborhoods with a high concentration of other foreign-born residents [32]. However, acute environmental stressors like the anti-immigration legislation and rhetoric that surrounded the 2016 US presidential election were associated with increased preterm birth among Hispanic women in the USA during that time period [33]. Thus, more attention to the importance of contextual and structural factors related to preterm birth is needed.

Living in neighborhoods of concentrated poverty and disadvantage is a contributing factor across all racial and ethnic groups to both incarceration and preterm births [7,34–36]. In fact, poverty is both a risk factor for and a consequence of incarceration [37–39]. Direct ICJS contributes to poverty due to collateral consequences such as loss of employment and educational opportunities, as well as the accumulation of fines and debt and strained social relationships [23]. Such collateral consequences broaden the impact of incarceration on the communities and families of incarcerated individuals return to [9,22,23,40]. Indirect ICJS is associated with poverty due to the economic strain and mental stress placed on the partners and families of incarcerated individuals [19,22,41]. Lastly, both incarcerated [17,42,43] and impoverished populations [44] bear a disproportionate burden of poor physical health, including infectious and chronic diseases, which in turn further increases reproductive risks. Thus, women in socioeconomically distressed communities can find themselves at a nexus of incarceration and poverty [42,44].

However, the relationship between socioeconomic factors and preterm birth is not as clear as it is between poverty and incarceration. For instance, the greatest disparity in Black–White preterm birth rates is found between US-born Black and White women of high socioeconomic status because Black women do not experience the same benefits of education and income as White women [7]. Additionally, neighborhood-level socioeconomic disadvantage contributes to preterm birth disparities through hazardous environmental exposures and chronic stress created by unemployment, over-policing, and violence [7]. However, the racial congruence of these neighborhoods also plays a role with Black women living in more economically stable neighborhoods that are predominantly White not experiencing the benefits their White neighbors gain from living in these neighborhoods [45].

Our county-level research contributes to this growing literature by examining preterm birth rates of women living within the nexus of poverty, mass incarceration, and rurality. Black Americans are more than twice as likely as White Americans to report having a family member or sexual partner who has been or is incarcerated, on probation, or on parole [19,20,23,42], and residents of low-income neighborhoods are at higher risk of indirect ICJS due to over-policing [34,36]. Additionally, almost one in three Black Americans born in the 1980s and 1990s report having an incarcerated loved one by 18 years old compared to less than 11% of Black “Baby Boomers” [19]. The mass incarceration of Black men has also resulted in Black women [36] disproportionately bearing the burden of paying court-related fees, leading parenting and household responsibilities, losing income, and supporting the needs of the incarcerated individual during and after their sentence [19,22,43,44]. This pervasive multilevel contact with the CJS throughout the lives of impoverished women, especially Black women, causes persistent exposure to stress [19,22,46], likely contributing to a physiologic cascade that negatively affects birth outcomes [47].

Investigation of the relationship between preterm births and county-level incarceration is of the utmost importance, as it provides an in-depth and nuanced narrative of national trends in birth outcome and criminal justice disparities. However, the investigation of the relationship between incarceration, preterm birth, and poverty is still a nascent field of study, and gaps remain in the literature. The purpose of this study was to examine the influence of county-level incarceration and poverty indicators on racial disparities in preterm birth in US counties of delivery. Our study tests the hypothesis that county-level jail admission rates in 2018, county-level economic distress, and rurality will be positively associated with preterm birth rates in the county of the delivery in 2019 and that the strength of these associations will be greater for Black women than for White or Hispanic women.

## Methods

### Data Sources, Sample Population, and Study Variables

We drew on multiple county-level data sources to create a comprehensive profile of county characteristics across 766 counties in 12 Southern and/or rural states in the USA (AL, AR, FL, GA, KY, LA, MO, MS, NC, SC, TN, TX, and WV). All datasets were merged using a five-digit Federal Information Processing System (FIPS) county identifier code. We used data from the CDC National Environmental Public Health Tracking System (NEPHT) to calculate preterm birth rates, which we defined as the percentage of live singleton births born before 37 completed weeks of gestation to Black, Hispanic, or White women who gave birth in that county in 2019 [48]. Pregnancies with multiple fetuses were not included due to higher risk of early delivery [49]. NEPHT data were originally sourced from the CDC Vital Statistics database, and estimates are derived from birth certificate data, which records the place of birth but not the home county of the person giving birth.

We used jail admission data available through the Vera Institute [50]. Importantly, we chose to use jail admission rather than prison admission data because many more individuals are impacted by jail incarceration – more people cycle through jails than prisons. We identified the number of all jail admissions for each county in 2018 and calculated the jail admission rate per 100,000 county residents using the total county population as the denominator, sourced from the American Community Survey [51]. However, the Vera dataset does not have demographic information, such as gender, race, or ethnicity. Vera data only have jail admissions in aggregate at the county level. Given that both the preterm birth and jail datasets are only available in annual increments, we included a lag of 1 year between jail admissions in 2018 and birth outcomes in 2019 to ensure that admissions preceded subsequent preterm birth. The purpose of the 1-year timeframe is to approximate a temporal order between jail admissions and births at the county level toward establishing a timeline of events.

We used the most recent Distressed Communities Index (DCI; pooled 2015–2019) to measure the overall level of economic stress for each county [52]. The DCI is a composite measure of seven indicators of economic stress: 1) percentage of adults 25+ years old without a high school diploma or equivalent; 2) percentage of prime-age adults (25–54 years) currently unemployed; 3) percentage of the population living below the poverty line; 4) median household income as a percentage of the surrounding metro or state median household income; 5) percentage of unoccupied habitable housing, excluding seasonal and recreational property; 6) percent change in the number of available jobs between 2015 and 2019; and 7) percent change in the number of business establishments between 2015 and 2019. The distress score ranges from 0 (minimal distress) to 100 (maximum distress).

Finally, we used the Index of Relative Rurality (IRR) to determine geographic differences between counties [53]. The IRR is a multidimensional, continuous, threshold- and unit-free composite measure of rurality and ranges on a scale from 0 (less rural, i.e., urban) to 100 (very rural). It takes into account four important dimensions of rurality, including the county population size, population density, remoteness (i.e., distance to the nearest metropolitan area), and built-up environment (i.e., extent of urbanized area as a percentage of total land area).

### Analysis

All analyses were performed with SAS software (v9.4). We generated summary statistics for each study variable, including the mean and standard deviation. We used linear regression to model the bivariate associations between premature birth and each independent variable. Because three premature birth values were recorded for each county (percentage of babies born to Black, Hispanic, or White mothers), we stratified our regression models by racial/ethnic group. We applied a log transformation to the Black, Hispanic, and White dependent variables of the percentage of births to achieve a normal distribution for use in linear models. Our transformed outcomes of the percentage of preterm births normally distributed (Black, Kolmogorov-Smirnov D-statistic [KS] = 0.12, *p* < 0.01; Hispanic, KS = 0.17, *p* < 0.01; White, KS = 0.19, *p* < 0.01), thus requiring no non-linear model specifications. We reported the F-value for the Type III fixed-effects solution for each independent variable to ease interpretation of results and comparability between variables and groups using a log-transformed outcome.

We used multivariable linear regression to model the percentage of preterm births, stratified by Black (Model 1), Hispanic (Model 2), and White (Model 3) mothers. Each model included all three independent variables of interest, including county-level jail admission rate in 2018, the DCI, and the IRR. Given the wide range of demographic, social, and economic county characteristics included in the composite DCI score, we did not adjust for additional county characteristics.

## Results

To determine if county-level jail admission rates in 2018, county-level economic distress, and rurality were associated with preterm birth rates in the county of delivery in 2019, we merged all five datasets to give complete data for 766 out of 1296 counties (59%) across 12 Southern/rural states in our sample. Table 1 provides county-level characteristics and bivariate associations. In brief, the mean percentage of babies born preterm was highest among Black mothers (13.0%), followed by Hispanic (9.0%) and White mothers (8.6%) (Table 1). The highest percentage of premature Black births was in Karnes County, TX (30%), the highest percentage of premature Hispanic births was in Monroe County, GA (19%), and the highest percentage of premature White births was in Newton County, MS (15%).

**Table 1.** County-level characteristics and bivariate associations (N = 766 counties)

			Type III Tests of Fixed Effects					
	Summary statistics		Black premature births		Hispanic premature births		White premature births	
Variables	Mean	STD*	*F*-value	*p*	*F*-value	*p*	*F*-value	*p*
Percentage of Black women’s babies born premature in 2019	12.96	1.99	–	–	–	–	–	–
Percentage of Hispanic women’s babies born premature in 2019	8.95	1.64	–	–	–	–	–	–
Percentage of White women’s babies born premature in 2019	8.63	1.19	–	–	–	–	–	–
Jail admission rate per 100,000 county adult residents in 2018	6,137.37	8,364.74	2.31	0.13	1.85	0.17	6.61	<0.0001
Distressed Communities Index (0–100)	54.08	28.99	54.50	<0.0001	1.70	0.19	82.17	<0.0001
Index of Relative Rurality (0–100)	47.02	8.39	19.35	<0.0001	2.63	0.10	74.38	<0.0001

* Standard deviation.

*Note:* Dependent variables for premature birth are log-transformed.

In bivariate analyses, economic distress and rurality were positively and significantly associated with preterm births among Black and White women (*p* < 0.0001 for each group). Jail admission rate was only positively associated with premature births among White mothers (*F* = 6.61, *p* < 0.0001). In the fully fitted stratified models (Table 2), economic distress was positively associated with premature births among Black (*F* = 33.81, *p* < 0.0001) and White (*F* = 26.50, *p* < 0.0001) mothers. Rurality was only associated with premature births among White women (*F* = 20.02, *p* < 0.0001). Jail admission rate was not associated with premature births among any racial group, and none of the study variables were associated with premature births among Hispanic mothers.

**Table 2.** Multivariable regression modeling the percentage of Black, Hispanic, and White women’s babies born premature in 2019

	Model 1 Black premature births		Model 2 Hispanic premature births		Model 3 White premature births	
Variables	*F*-value	*p*	*F*-value	*p*	*F*-value	*p*
Jail admission rate per 100,000 county adult residents in 2018	0.05	0.83	1.16	0.28	0.65	0.42
Distressed Communities Index (0–100)	33.81	<0.0001	0.15	0.70	26.50	<0.0001
Index of Relative Rurality (0–100)	0.35	0.55	1.03	0.31	20.02	<0.0001

*Note:* Dependent variables are log-transformed.

## Discussion

Here, we used the most up-to-date county-level secondary data sources to test our hypothesis that county-level jail admission rates in 2018, county-level economic distress, and rurality will be positively associated with premature birth rates in the county of the delivery in 2019 and that the strength of these associations will be greater for Black women than for White or Hispanic women in a sample of Southern US counties. Our examination failed to support several aspects of our hypothesis. First, jail admission rates were not significantly associated with preterm birth for White, Black, or Hispanic women in the fully fitted stratified models. Rurality only maintained a significant association with preterm birth among White women after controlling for other variables. Next, consistent with previous findings, preterm birth rates for Hispanic women were nearly identical to those for White women [5], and Hispanic preterm birth rates were not associated with any of the variables in our final fitted model. Lastly, in partial support of our hypothesis, DCI scores were positively associated with preterm births among Black and White women, and the relationship was strongest among Black women.

Research generally supports a positive association between incarceration rates and risk of preterm birth among Black mothers [54–58], with one study even finding that county-level racial inequity in jail incarceration was negatively associated with infant mortality among White populations [55]. In a large, nationwide epidemiologic study of data collected between 1999 and 2015, Jahn et al. (2020) found that preterm birth among Black and White women was positively associated with county-level jail incarceration rate [56]. The authors did not investigate trends among Hispanic women, and the statistically significant, yet modest, effect size may have been a function of their very large sample (over 40 million individual health records) [56]. While the lack of association between jail admissions and preterm births in our multivariate analysis was surprising, it is not fully inconsistent with the literature. Wallace et al. (2017) found that states with lower overall imprisonment rates had lower overall infant mortality rates, but this relationship was absent after considering confounding variables such as the ratio of Black to White imprisonment and the race-specific imprisonment rates [59]. Sealy-Jefferson et al. (2020) used data from a sample of Black women in California to test the predictive power of incarceration rate on risk of preterm birth up to 2 years later and failed to identify a significant relationship between preterm birth and incarceration rate [60].

Measurement variation across studies and limitations in stratification across databases make inconsistencies in research findings inevitable and replication of findings difficult. In the context of our study, there were several areas in which measurement decisions may have resulted in inconsistent results. We measured preterm birth because of its frequency in the population, its convincing linkage to stress-induced biophysiological pathways, and its strong association with other birth outcomes, as well as the development of chronic conditions later in life [5,6]. However, other studies on racial disparities in birth outcomes used different outcomes, including low birthweight for gestational age and infant mortality. Additionally, the area unit used to assess preterm birth varies widely. We only included counties that provided data for Black, Hispanic, and White women so that county-level comparisons could be made and to generate findings that could readily inform policy. To our knowledge, only two other studies used higher-level measures of both incarceration and birth outcomes [61,62]. Conway et al. (2021) used state-level prison incarceration rates to examine multiple state-level child health and birth outcomes; they found that annual state prison incarceration rates predicted infant mortality rate, preterm birth rate, and low birthweight rate the next year among all children born in that state, and the relationship was stronger for infant mortality and low birthweight for Black children than White children [61]. However, our use of jail admission data is an important distinction because many more people cycle in and out of jails than prisons [63]. Similarly, Wildeman (2012) used both state-level infant mortality rate and prison incarceration rate data collected between 1990 and 2003 and found that incarceration rate was positively associated with infant mortality [62]. Future research must strive to use the area unit that is ethically sound, reflective of how the population of interest defines their community, and appropriate for the underpinning theoretical framework [64].

Additionally, despite the fact that ICJS impacts health, contextual data on ICJS are limited. Thus, there is no consensus on how to comprehensively measure ICJS [65]. Direct ICJS has been measured with multiple variables across ecological levels. Given variation in the length of time, environment, healthcare resources, staffing, and criminal histories associated with each type of imprisonment facility, it is likely that prisons, jails, and juvenile custody may each have a different relationship with investigated health outcomes, including preterm birth. Similarly, indirect ICJS has been measured at an individual level as family, spouse, or household member who has been involved in one of the direct ICJS measures above across the lifespan. Indirect ICJS has also included exposure to neighborhood- (e.g., policing) or county/state-level factors (e.g., rates of direct ICJS among specific populations), all of which increase the risk of ecological fallacy when interpreting study findings. Currently, there is a lack of integrated data sources, and there should be more focus and emphasis on collecting quality data across the criminal justice continuum and integrating data across various timepoints and systems.

The DCI is unique in its assessment of seven socioeconomic indicators to contextualize economic conditions of US counties. We found a strong association between higher DCI score and preterm birth for Black and White women during multivariate analysis. Our results are consistent with numerous studies that found strong associations between preterm birth and individual and community-level measures of socioeconomic status [66]. However, social environment alone did not explain racial differences in preterm births because the association was identified for both Black and White women but not for Hispanic women. Additionally, prior research found that Black–White disparities in birth outcomes persist and sometimes widen when comparisons are made between women at higher socioeconomic and educational levels [67]. Consequently, noted health-disparities researchers hypothesize that Black women experience fewer of the health benefits associated with education and income than White women due to their inescapable exposure to structural racism and sexism [68–70]. These studies support the need for approaching the subject with an intersectional lens [71,72].

One underexplored piece of this structural intersectional framework is the effect of “place,” particularly the effect of rural–urban differences on racial preterm birth disparities. Despite the uniqueness of the lived experiences of rural Black women [73], their voice has been largely left out of research on preterm birth [74]. In our study, the rurality of the county where a woman gave birth was significantly associated with preterm birth among White women in both bivariate and multivariate analysis. However, among Black women, rurality was significant in bivariate analysis but no longer after accounting for county-level economic distress and incarceration. Our race-stratified finding suggests that rurality, which is strongly related to healthcare access [75], may explain more of the risk of preterm birth for White women than for Black women. This finding is not entirely surprising, with the Black–White disparity in preterm birth persisting despite closing gaps in prenatal and preconception healthcare between Black and White women in the USA [7].

The adverse effect of rurality on preterm birth is related to the fact that rural areas experience greater shortages of healthcare providers and have fewer preventative health and obstetric services [30,75,76,77]. Our findings suggest that White women giving birth in rural counties may not have access to the same advantages as their White urban counterparts. Similarly, White women giving birth in counties with lower economic well-being may have fewer economic resources than White women in more prosperous counties. Consequently, White women in both scenarios would have access to fewer health-benefitting resources and therefore have a higher risk of preterm birth. In a race-stratified meta-analysis, White women in the most disadvantaged areas had 48% higher odds of preterm birth than White women in the least disadvantaged areas, while Black women in the most disadvantaged areas had only 15% higher odds of preterm birth than Black women in the least disadvantaged areas [66]. Ultimately, much of this work points to preterm birth disparities between Black and White women being caused by structural racism against Black Americans, regardless of their location or socioeconomic status [7].

Although examination of racial disparities in rural areas is limited, our finding that rurality was not associated with preterm births among Black women is not unique in the literature. Despite consistently having fewer educational and economic resources and less access to healthcare than White rural residents, Black rural residents do not have unilaterally poorer health outcomes or worse health behaviors than White rural residents [31,76,77]. Consequently, we propose another potential explanation for our findings from a strengths-based intersectional perspective. Perhaps Black women living in rural areas, especially those in racially congruent rural areas, have access to culture- and region-specific advantages that buffer deleterious health effects; such advantages include increased access to social support, resource sharing, and social capital [45]. As rural America becomes increasingly diverse [78], future research should investigate urban–rural differences in racial health disparities and the measurement of structural racism, sexism, and economic inequality within rural populations. This is especially true within regions that have unique social, political, and historical contexts such as the US South, where more than 93% of rural Black Americans reside [31,45,76,77]. Future research should also use qualitative and mixed-methods research to identify and explore differential utilization of protective factors by rural women across diverse racial and ethnic groups.

### Limitations

Our study had limitations related to the analysis of five secondary datasets that must be accounted for when interpreting our findings, including the unidimensional measurement of race, jail incarceration, and rurality. First, the potential for confounding at the county level must be acknowledged, such that some county characteristics may be related to both our outcome (preterm birth) and our predictors (jail admissions and economic distress). To address this, we used a composite score of economic distress derived from multiple social and economic determinants of health, including education, employment, poverty, income, housing, jobs, and business. As a result, we eliminated the problem of highly correlated predictors and simultaneously incorporated contextual factors into the study design. Additionally, because risks and exposures vary widely between urban and rural areas, we adjusted our models to include a dynamic measure of rurality.

Second, the use of birth certificate-derived data from the NEPHT database limited us to investigating preterm birth rates based on county of delivery and not the county of residence. Future studies should evaluate the community environments where pregnant women live, as those environments may differ from where the baby was actually delivered. This is particularly true for pregnant women living rural counties because they are more likely to give birth outside their home county due to a lack of healthcare services. We adjusted our model for rurality to help parse out this disparity, but it is still an important limitation. To our knowledge, there are no national or regional publicly available datasets that have individual-level information about birth outcomes and other individual characteristics. This is an important limitation of national and regional CDC surveillance data and has implications for how we interpret county-level findings.

Like the NEPHT, the most recent Vera county-level jail admission data do not include individual-level data such as race, ethnicity, gender, and residence; it only includes aggregate information about the total number of jail admissions for a given county where individuals were incarcerated. While we acknowledge this is an important limitation, the USA has more than 3000 counties, most of which have their own jail facility, making it untenable to contact each of these jails to obtain demographic data. Despite its limitations, the Vera dataset is, to our knowledge, the only publicly available national incarceration dataset, which makes building collaborations with local community-based partners involved in the criminal justice system especially important to advance this field of research. Future research should apply the principles of community-based participatory research to build such collaborations [79].

Despite the limitations, the scope of our county-level study provides an epidemiologic narrative of disparities that may inform the design of future multilevel studies. Strengths of the study were the use of a novel measure of economic well-being and our accounting for shared historical context by analyzing data from counties in Southern and rural states. Additionally, our inferences about the risk of preterm birth among women in the US South were based on analytic results from aggregate, county-level variables collected from these secondary databases. However, county-level measurement may mask nuanced differences between neighborhoods, requiring more granular assessment than even zip codes or census tracts offer [64]. For instance, Salahuddin et al. (2022) found that infant mortality and maternal risk factors varied based on zip code in two Texas counties. Identification of geographic variation at this smaller area unit level resulted in critical adaptations to the implementation of pregnancy-related services [80]. Thus, Hardeman et al. (2022) recommend that researchers align their examined geographic context with appropriate theory and research questions [64]. However, alignment can be difficult when state-level data are recommended for informing policy decisions [81], county-level data are subject to gerrymandering and redistricting, and neighborhood-level data may be unavailable [64]. Future research should use theory and community input to determine which level is most appropriate.

## Conclusion and Future Research Directions

Our study identified differential associations between economic distress and preterm birth, with notable concentrations of high poverty and high preterm birth rates in the US South. Despite the USA far outpacing other countries in healthcare expenditures, Black women in some of the counties we studied had preterm birth rates that were higher than the rates found in many low-income countries [3]. Regardless of location, the financial, emotional, and social consequences of preterm birth are incalculable. Therefore, we must investigate all avenues by which to explore contributors to this public health tragedy. In the context of translational research, the relationship between preterm birth, incarceration, and racial disparities is not straightforward, and findings are mixed. Disentangling and understanding the complex reinforcing relationships that connect health outcomes to enduring structural inequities is a daunting, yet necessary, endeavor.

Our study contributes to literature aimed at informing policy-making and programmatic decisions regarding the deleterious health effects of the social and economic determinants of health associated with community distress. Our findings echo the importance of investing in the economic well-being of distressed communities to affect downstream health outcomes, including preterm birth. Future research should examine and test ways that community-level interventions can improve economic and structural environmental factors to influence preterm birth rates. Future research should also use mixed methodological approaches to conduct in-depth investigations of the influence of distressed community environments on pregnancy experiences and diverse exposures to the criminal justice system across the life course of Black and White women in the South. Given mixed findings in the extant literature, both qualitative and quantitative data are needed to elucidate the true relationship between incarceration and preterm birth at key stages of a woman’s reproductive life cycle. Future research should be informed by interdisciplinary theoretical frameworks that will inform the conceptualization and measurement of indicators of structural racism. Lastly, future studies should continue to examine, at appropriate population levels, the relationship between preterm birth and socioenvironmental factors, including those accounted for in the DCI. Although not perfect, the identification of specific factors driving this strong association can inform the development and testing of translational health-disparities interventions in the future.
